# Divergent effects of successive drought and flooding on photosynthesis in wheat and barley

**DOI:** 10.3389/fpls.2025.1603355

**Published:** 2025-08-25

**Authors:** Shukanta Saha, Giles N. Johnson

**Affiliations:** ^1^ Department of Earth and Environmental Sciences, Faculty of Science and Engineering, University of Manchester, Manchester, United Kingdom; ^2^ Department of Botany, Faculty of Life and Earth Sciences, Jagannath University, Dhaka, Bangladesh

**Keywords:** drought, flooding, photosynthesis, wheat, barley

## Abstract

Climate change is leading to increases in extreme weather events, notably increasing both droughts and floods, which undermine food security. Although each stress individually has been well studied, little is known about the response of cereals to successive water stresses, condition that often occurs in real-world scenarios. To address this gap, we have compared physiological responses of wheat and barley cultivars to cycles of drought and flooding. We show that these species show different responses to each other and that successive stresses result in different responses to single stresses. Plants were subjected to control, drought or flooding treatments for 15 days. Following that, previously stressed plants were exposed to a further stress – drought followed by flooding (D-F) or flooding followed by drought (F-D) for a further 15 days. These cereals showed contrasting responses both to drought or flooding alone and to successive stresses (D-F or F-D). Barley retained photosynthetic capacity when exposed to single- drought or flooding, whereas wheat responded to both stresses with significant declines in CO_2_ assimilation capacity by 41% and 31% in response to drought or flooding, respectively -primarily due to stomatal closure. However, the first exposure to water stress impacts the inhibition of photosynthesis during subsequent stress. The effect of subsequent water stress – drought or flood –was continued and aggravated by the previous stress in wheat. Importantly, non-stomatal factors were induced, which reduced Photosystem II efficiency (62% and 49%) and chlorophyll content (35% and 47%) in wheat under D-F and F-D stress. By contrast, barley retained its photosynthetic capacity under D-F stress by acclimating, with 41% reduced shoot growth, while F-D treatment induced abnormal stomatal development. Both treatments resulted in the accumulation of carbon in tissues. Overall, we conclude that sensitivity to a stress is increased by the exposure to a previous stress, with F-D stress having the largest effect, while barley is relatively more tolerant than wheat highlighting it as the more robust cereal crop under fluctuating water conditions.

## 1 Introduction

Climate change is increasing the frequency and severity of drought and flooding events, hampering food security. Drought affects plants throughout all developmental stages by impairing key physiological processes including photosynthesis (Hossain et al., 2012; Kilic and Yagbasanlar, 2010). The loss of photosynthesis can lead to reduced growth and ultimately seed yield (Ahmad et al., 2022; Chen et al., 2012; Ahmad et al., 2018). Conversely, flooding affects approximately 10% of arable land (Yaduvanshi et al., 2012) and causes a reduction of oxygen levels in the soil with hypoxic conditions created. Flooding increased the accumulation of CO_2_ and ethylene that disrupts the normal physiological and morphological development of plants (Kreuzwieser et al., 2004). The severity of flooding depends on factors such as duration, developmental stage and species or cultivar characteristics (Manghwar et al., 2024). Key effects include reductions of photosynthetic rate (P_n_), transpiration (E) and water use efficiency (WUE), along with damage to the photosynthetic apparatus, particularly during early growth stages (Shao et al., 2013). Flooding leads to a loss of chlorophyll in any growth stage (Chen et al., 2005). Stomatal closure is a primary response to both drought and flooding that leads to decrease the rate of net photosynthesis and under severe water stress conditions, metabolic impairment maybe added to the stomatal limitation. At progressive stages of water stress, stomatal traits such as size, density and distribution can be altered (Pirasteh-Anosheh et al., 2016; Zhou et al., 2020).

Climate change forecasts predict increasing frequency of both drought and flooding events across cereal growing region (Borowski, 2020; Eckstein et al., 2020). Importantly, plants are increasingly exposed to these stresses successively at the same growth phase, and yet, their responses to such sequential stresses are complex and not simply additive. Evidence suggests that the physiological responses to alternating drought and flooding are distinct from single drought or flooding responses (Rivero et al., 2022; Walter et al., 2013; Biswas and Jiang, 2011). For example, drought followed by flooding (D-F) induced photosynthetic, growth and yield reductions in rice (Xiong et al., 2018; Zhu et al., 2020), and cabbage (Barber and Müller, 2021). Qian et al. (2020) showed that all yield indices of cotton were reduced by flooding followed by drought (F-D), but the reduction was lower than the additive effects of both stresses imposed separately. Winter flooding followed by summer drought reduced yield in wheat, with this effect being additive but not more than additive (Dickin and Wright, 2008). However, a research gap remains regarding species-specific responses of major cereals like wheat and barley to successive alternating water stresses, particularly at physiological and biochemical levels.

Wheat and barley are important cereal crops, directly or indirectly providing a significant part of the calories in human diets globally. With expanding global populations, food security is becoming increasingly difficult, especially with unpredictable severe weather patterns (Wheeler and Von Braun, 2013). Water stress is critical for wheat production and this is a serious problem, as wheat is the staple food of 35% of the world population (Ostrowska and Hura, 2022; Soares et al., 2019; Tadesse et al., 2019). Barley is the fourth most important cereal crop (Giraldo et al., 2019) and is regarded as being better adapted to drought stress (Dawson et al., 2015). The high amount of genetic variability towards stress tolerance has led to its use as model plant in studying water use efficiency (Ostrowska and Hura, 2022).

Despite many studies published on separate drought and flooding responses in wheat and barley, little is known about the physiological or biochemical traits resulting from cycles of drought and flooding in these important cereals. Such knowledge is necessary to identify novel traits conferring resilience and for cereal crop improvement to withstand sequential water stresses. The present study aims to investigate the physiological responses of wheat and barley to cycles of drought and flooding, with single drought or flooding and drought followed by flooding (D-F) or flooding followed by drought (F-D) treatments, as well as their recovery responses. We have focused on identifying physiological features that give rise to cyclical drought-flooding stress tolerance. To achieve this, we have focused on photosynthesis, chlorophyll content and growth parameters as photosynthesis is essential for plant growth and yield and its stability under water stress can indicate better physiological resilience. We hypothesize that successive applications of drought and flooding (D–F and F–D) will lead a greater inhibition of photosynthesis than either drought or flooding alone. Furthermore, we predict that barley will exhibit greater physiological resilience to these cyclical stress conditions than wheat, as evidenced by better photosynthetic performance.

## 2 Materials and methods

### 2.1 Plant growth conditions and stress treatments

**Figure 1:**
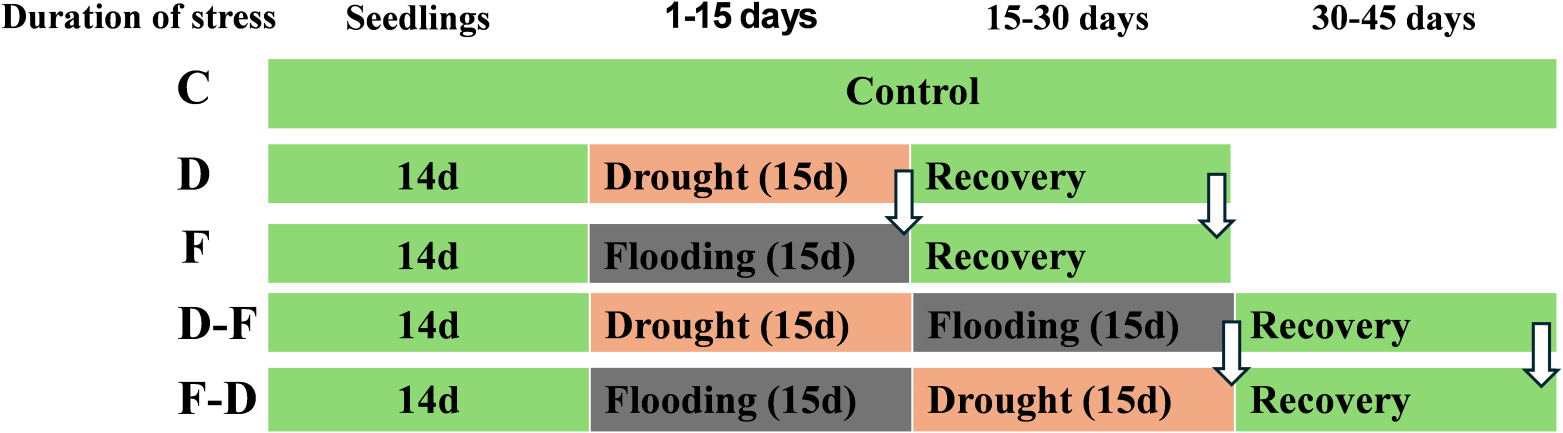
A diagram illustrating experimental set up and stress treatments applied in wheat and barley. Two-week (14 days) old plants were subjected to control, drought or flooding for 15 days. Following that, previously stress-treated plants were exposed to a further stress – drought followed by flooding (D-F) or flooding followed by drought (F-D) for a further 15 days. For recovery treatments, plants were returned to normal irrigation for 15 days. Arrows indicate measurement at the end of single and successive stresses as well as recovery. C, control; D, drought; F, flooding; D-F, drought followed by flooding; F-D, flooding followed by drought.

Plants were grown in a growth room at a light intensity of 100 μmol.m^-2^.s^-1^, provided by warm white LED tubes (color temperature 2800–3200 K), 20°C/16°C day/night temperature, 16/8h day/night photoperiod and relative humidity (RH) 45-50%. Gleam Wheat (*Triticum aestivum* L.) and Chariot Barley (*Hordeum vulgare* L.) cultivars seeds were grown at 7-inch plastic pots in peat-based multipurpose compost. The plants were grown for two-weeks and treatments were imposed at the 3-leaf stage. Soil moisture content (SMC) was maintained at 80% in control and 30% in drought treatment. Flooding treatment was imposed by placing pots in water-filled containers (42 cm X 28 cm X 12 cm) where water was maintained 2 cm above the soil surface. 14 days-old plants were subjected to drought or flooding treatments for 15 days. Following that, previously stress treated plants were exposed to a further stress – drought followed by flooding (D-F), or flooding followed by drought (F-D) for a further 15 days. For recovery treatments, plants were returned to normal irrigation for 15 days (**Figure 1**). The selection of the drought and flooding treatments was intended to focus on acclimatory responses rather than lethal effects, so we chose 30% SMC in drought and 2 cm water level in flooding treatments as a moderate stress level and a 15-day treatment duration was selected for both drought and flooding conditions to ensure consistent and measurable stress exposure without causing irreversible damage, thereby enabling the assessment of plant acclimation and recovery.

In drought experiments, plants were allowed to dry by withholding water until 30% SMC was reached (12 days taken) and SMC was measured everyday throughout the stress period. This soil moisture content was then maintained by rewatering daily. Soil moisture content (SMC) is expressed as a percent maximum soil water holding capacity. To estimate soil water holding capacity, 1.1 kg soil in pots of fully water-saturated soil were weighed and then dried to constant weight at 90°C. The weight difference between water saturated and oven dried soil was taken as weight of water needed to bring pots to soil water holding capacity and lower SMCs (% water holding capacity) were calculated accordingly. Flooding treatment was also imposed at two weeks-old plants by placing pots in big water filled container (42 cm X 28 cm X 12 cm) where water was maintained 2 cm upper from the soil surface of the pot.

### 2.2 Growth analysis

At harvest, fresh roots and shoots were separated and dried to constant weight in an oven at 70°C.

For specific leaf area (SLA) measurement, leaf pieces were scanned using a flatbed scanner and their area determined using ImageJ image analysis software (National Institutes of Health, USA). Tissues were subsequently dried in an oven at 70°C for 72 h and weighed to determine the dry weight. Specific leaf area (SLA) was calculated as leaf area divided by leaf dry weight using the same leaves used as for gas exchange and chlorophyll fluorescence measurements.

For measurements of relative water content, leaves were excised and weighed immediately to estimate fresh weight (FW). Then, they were floated on the distilled water in a falcon tube and left at room temperature for 24 hours. The leaves were then re-weighed to estimate turgid weight (TW), after which they were dried for 48h h in an oven at 70°C to obtain dry weight (DW) as described by Boyer et al. (2008). RWC was calculated following the equation: RWC (%) = (FW – DW)/(TW – DW) x 100, where RWC is expressed as a percentage of the fully turgid water content (TW – DW).

### 2.3 Measurement of gas exchange and chlorophyll florescence parameters

Gas exchange parameters were measured using an LI-6400XT infrared gas analyzer (LI-COR, NE, USA) following the protocol of Johnson and Murchie (2011). Whole plants were dark adapted for 30 minutes before starting measurements under a saturating PPFD (photosynthetic photon flux density) of 1000 μmol m^-2^s^-1^ light intensity (red: blue 90: 10) and ambient CO_2_ (400 μl l^-1^). Fully expanded 4^th^ leaves were clamped into a LiCor extended reach leaf chamber and assimilation in the dark was recorded after allowing the leaf to equilibrate in the IRGA chamber for 10 min and assimilation in the light and transpiration were recorded at steady state, after 20 min exposure to light. Chlorophyll fluorescence parameters were measured during gas measurement under the same conditions. PSII efficiency (ϕPSII) and non-photochemical quenching (NPQ) were estimated as described by Maxwell and Johnson (2000).

### 2.4 Measurement of stomatal density and size

Leaves used for gas exchange measurements were taken for the measurement of stomatal density and size and 3–5 points were selected either side of the middle of the leaf, avoiding the tip and base. A layer of clear nail varnish was applied on the adaxial surface of the leaves and allowed to dry. A second layer of nail varnish was then applied and left to dry. A pair of fine forceps was used to grip the varnish which was then placed on a microscope slide. After adding two drops of distilled water, a coverslip was placed over the varnish and the sample was observed under a light microscope at 10X magnification with camera (GXML 2800, UK). The size of field of view was estimated with a standard microscopic 1 mm scale and, after counting stomata from photographs, it was converted to stomatal density per mm^2^ of leaf area. Stomatal size was measured using ImageJ.

### 2.5 Determination of chlorophyll content

A leaf segment (~ 50 mg) was weighed and ground with a mortar and pestle containing 80% v/v acetone freshly prepared. The extract was made to 5 mL with acetone. After mixing, 1 mL of solution was transferred to a centrifuge tube and made to 2 mL with 80% acetone and then centrifuged it at 15000 rpm for 5 minutes. Then, the supernatant was placed in a glass cuvette for absorbance measurement using an Ocean Optic USB4000 spectrophotometer and chlorophyll content calculated according using the method of Porra et al. (1989). The values obtained were converted to nmol/mg fresh weight of leaf.

### 2.6 Nitrogen and carbon content determination

Plants were harvested at the end of the first stress treatment (Day 15), following further stress or recovery (Day 30) and following recover from double stress treatments (Day 45). Harvested plant were separated into roots and shoots. Sub-samples were oven dried at 70°C for 72 h and subsequently weighed for determination of dry mass. Dried tissues were finely ground with ball mill grinder (Retsch, Haan, Germany). Ground sub-samples were loaded into tin capsules (5mg sample) with measuring micro-balance and acetanilide used as standard. Then, samples as well as standards were dry combusted in a CHN elemental analyzer (Vario EL CUBE Elemental Analyzer) in the presence of helium as a carrier gas to determine N and C content.

### 2.7 Statistical analysis

Statistical analysis was done using GraphPad Prism (version 9.3.1) software. Two-way ANOVA and one-way ANOVA followed by Tukey test for multivariate analysis (p < 0.05) were applied as indicated in figure legends. Data shown are the mean ± SE of 5 biological replicates. Different letters above the bars show significant differences at p < 0.05.

## 3 Results

### 3.1 Growth analysis under cycles of drought and flooding in wheat and barley

Two-week-old wheat and barley plants were subjected to control, drought or flooding treatments for 15 days. Following that, previously stress-treated plants were exposed to a further stress – drought followed by flooding (D-F) or flooding followed by drought (F-D) for a further 15 days. For recovery treatments, plants were returned to normal irrigation for 15 days. Plants were harvested at the end of the first stress treatment (Day 15), following further stress or recovery (Day 30) and following recovery from double stress treatments (Day 45).

**Figure 2:**
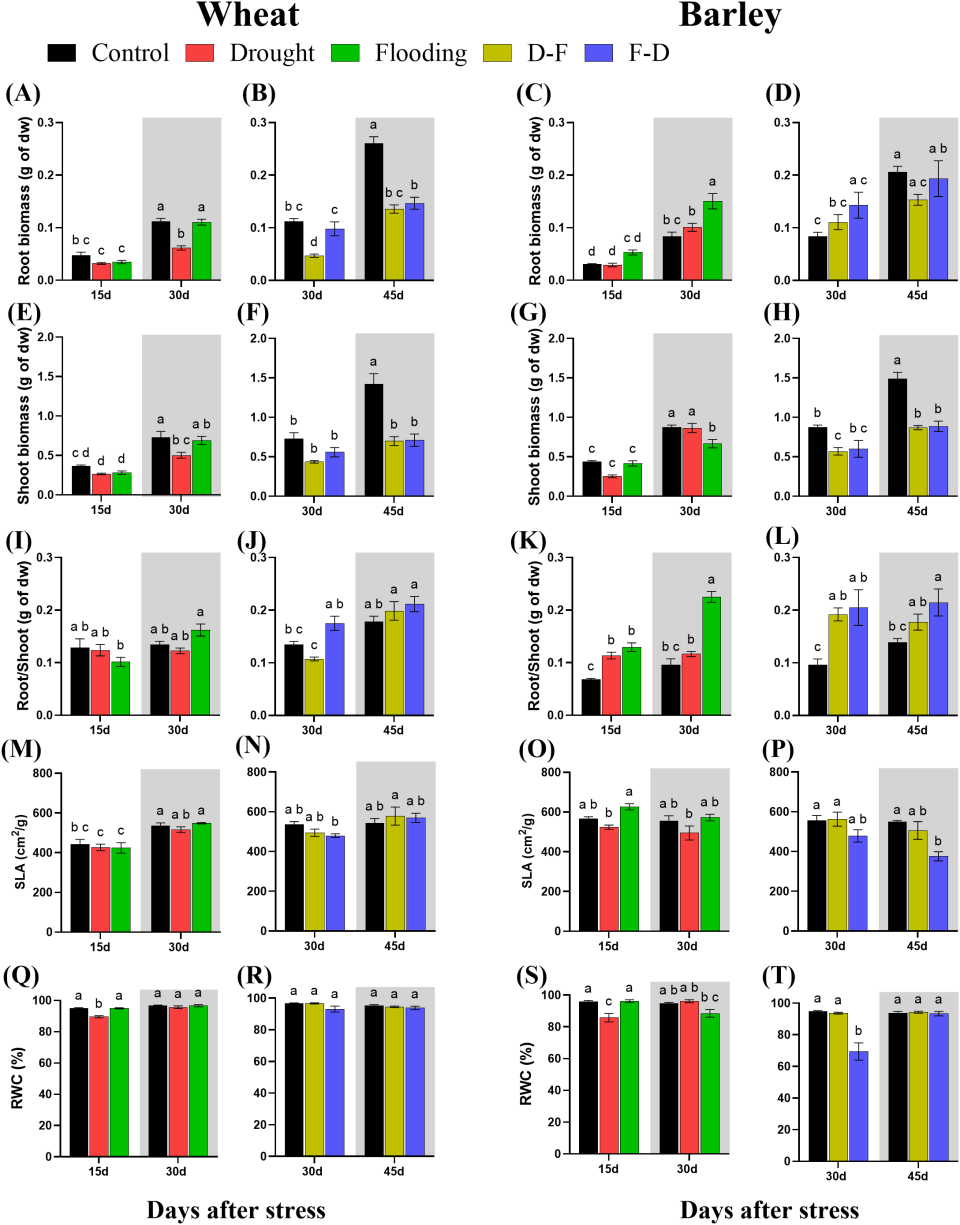
Root dry biomass **(A–D)**, shoot dry biomass **(E–H)**, root/shoot **(I–L)**, specific leaf area [SLA; **(M–P)**] and leaf relative water content [RWC; **(Q–T)**] under cycles of drought and flooding in wheat and barley. Grey areas correspond to recovery. Data are the mean ± SE of 5 biological replicates. Different letters above the bars show significant differences at p< 0.05 (two-way ANOVA/Tukey *post hoc* test). C, control; D, drought; F, flooding; D-F, drought followed by flooding; F-D, flooding followed by drought.

In wheat, exposure to either drought or flooding for 15 days did not immediately affect root or shoot growth significantly (**Figures 2A, E**). After a 15-day recovery period following a single stress, shoot and root dry biomass were reduced in previously droughted plants (by 50 and 58% respectively), though no reduction was seen in the flooded plants (**Figure 2A**, grey area; 2E, grey area). Root:shoot ratio was unaffected by either treatment (**Figure 2I**; **Table 1**). In barley, root biomass at the end of the stress was unaffected in droughted plants but increased significantly (P= 0.0003) by 79% in flooded plants (**Figure 2C**). At the same time, shoot biomass was significantly decreased following recovery from flooding (**Figure 2G**; grey area). As a result, the root:shoot ratio of barley plants was significantly (P< 0.0001) increased by 1.3- fold following flooding (**Figure 2K**, grey area; **Table 1**). A two-way ANOVA results for root/shoot ratios in barley showed a highly significant difference between treatments and days, along with their interactions (**Table 1**).

**Table 1:** Results (F and P values) of two-way ANOVA of growth of wheat and barley under cycles of drought and flooding.

Traits	Wheat				Barley			
	Single stress		Successive stress		Single stress		Successive stress	
	F	P	F	P	F	P	F	P
Root biomass								
Treatments	28.94	**<0.0001**	41.43	**<0.0001**	14.87	**0.0001**	1.734	0.1998
Days	219.2	**<0.0001**	128.7	**<0.0001**	108.1	**<0.0001**	19.71	**0.0002**
Interaction	13.92	**0.0003**	11.23	**0.0006**	3.339	0.0561	2.448	0.1097
Shoot biomass								
Treatments	7.502	**0.0046**	21.86	**<0.0001**	4.289	**0.0282**	32.50	**<0.0001**
Days	86.67	**<0.0001**	29.37	**<0.0001**	158.3	**<0.0001**	58.22	**<0.0001**
Interaction	2.025	0.1626	5.863	**0.0095**	9.591	**0.0012**	4.139	**0.0298**
Root/Shoot								
Treatments	0.3745	0.6924	6.049	**0.0088**	75.47	**<0.0001**	13.33	**0.0002**
Days	5.448	**0.0301**	29.18	**<0.0001**	42.26	**<0.0001**	0.7134	0.4079
Interaction	4.159	**0.0309**	2.685	0.0927	19.29	**<0.0001**	1.341	0.2831
SLA								
Treatments	0.4917	0.6181	0.2903	0.7515	9.676	**0.0014**	8.625	**0.0029**
Days	42.11	**<0.0001**	11.81	**0.0029**	3.249	0.0882	4.382	0.0526
Interaction	0.4415	0.6486	0.2903	0.7515	0.5250	0.6003	1.058	0.3703
RWC								
Treatments	23.26	**<0.0001**	3.013	0.0743	3.842	**0.0378**	10.89	**0.0009**
Days	50.69	**<0.0001**	1.278	0.2732	0.1766	0.6786	8.946	**0.0082**
Interaction	10.25	**0.0007**	0.9158	0.4181	15.30	**<0.0001**	9.934	**0.0014**

Bold P values indicate significant differences at P< 0.05.

In wheat, D-F treatment reduced root biomass relative to plants maintained in control conditions, whilst F-D treatment did not significantly affect this (**Figure 2B**). Shoot biomass was unaffected at the end of either treatment (**Figure 2F**), however following a recovery period, both root and shoot biomass were significantly reduced in both treatments (**Figure 2B, F**, grey area; **Supplementary Figure S3**; **Table 1**), with the root:shoot ratio being unaffected (**Figure 2J**, grey area). By contrast, in barley both sequential treatments resulted in reduced shoot biomass following recovery with 41% and 40% loss at D-F and F-D respectively (**Figure 2H**, grey area), but with no significant effect on roots, resulting in an increased root:shoot ratio (**Figure 2L**).

Specific leaf area (SLA) was not changed depending on treatment in wheat (**Figures 2M, N**). In barley, flooding increased SLA as compared to drought stressed plants (**Figure 2O**) whilst F-D treatments caused a significant decrease in SLA after the stress, Day 45 (**Figure 2P**).

Leaf RWC was not affected by any stress treatment in wheat, except drought at Day 15 (**Figure 2Q**). A similar response was seen in barley, where drought decreased leaf RWC (**Figure 2S**). Exposure to drought following a flooding stress (F-D) substantially reduced leaf RWC in barley leaves, however plants recovered following a return to normal watering conditions (**Figure 2T**).

### 3.2 Photosynthetic responses to cycles of drought and flooding in wheat and barley

**Figure 3:**
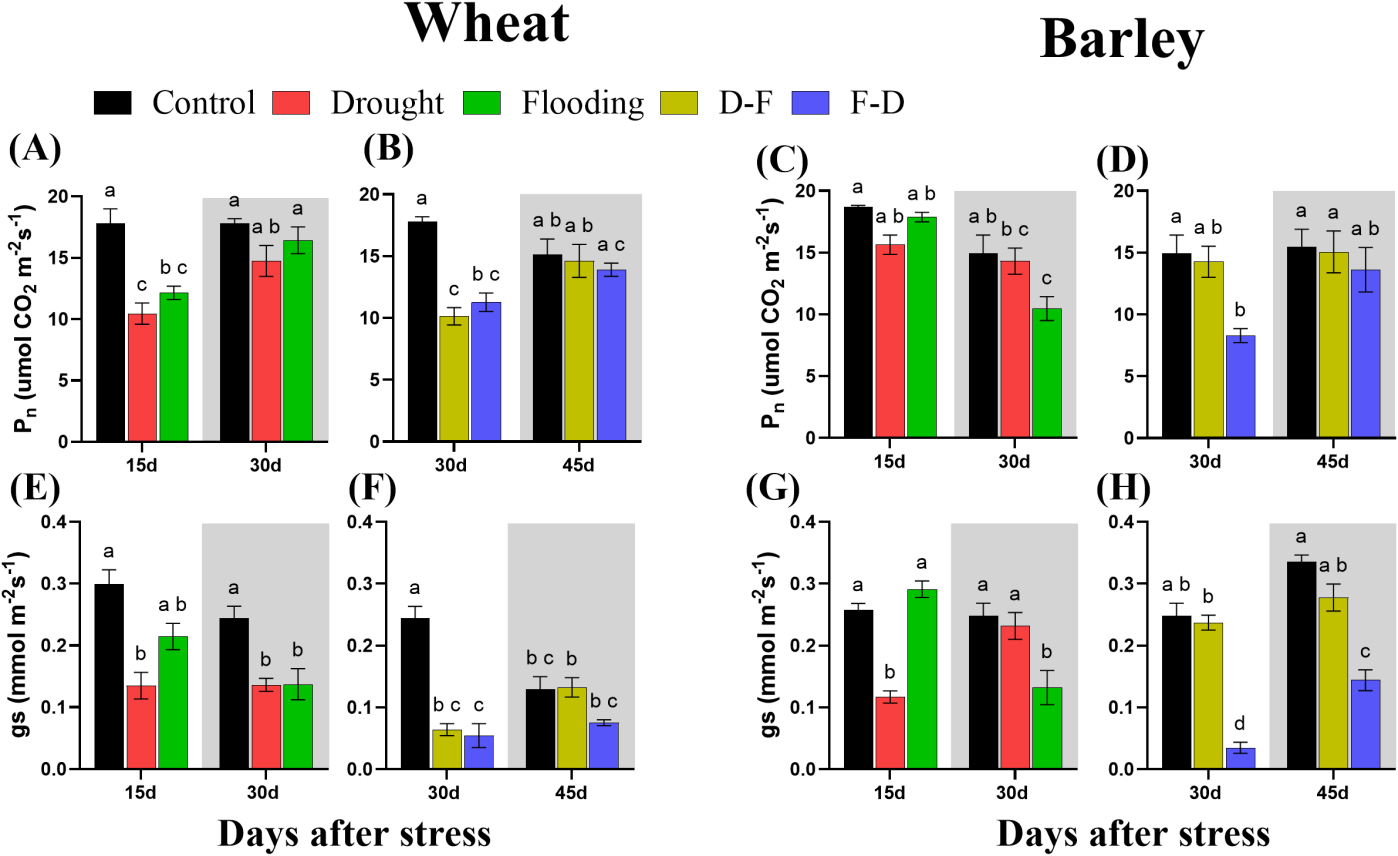
Net photosynthetic rate (P_n_; **(A–D)**) and stomatal conductance [g_s_; **(E–H)**] under cycles of drought and flooding in wheat **(A, B, E, F)** and barley **(C, D, G, H)**. Grey areas correspond to recovery. Two-week-old wheat and barley plants were subjected to control (80% SMC), drought (30% SMC) and flooding individually for 15 days, drought followed by flooding (D–F) and flooding followed by drought (F–D) for 30 days. For recovery treatment, plants were returned to normal irrigation for 15 days. P_n_ was measured as the net rate of photosynthesis at an irradiance of 1000 μmol m^-2^s^-1^ and ambient CO_2_ (400 µl l^-1^) and the g_s_ data were taken during measurements of P_n_. Data are the mean ± SE of 5 biological replicates. Different letters above the bars show significant differences at p< 0.05 (two-way ANOVA/Tukey *post hoc* test). C, control; D, drought; F, flooding; D-F, drought followed by flooding; F-D, flooding followed by drought. SMC, soil moisture content.

The photosynthetic performance of plants was measured at the end of the first stress treatment (Day 15), following further stress or recovery (Day 30) and following recovery from double stress treatments (Day 45) (**Figure 3**). In wheat, the light-saturated net photosynthetic rate (P_n_) decreased significantly (P= 0.0005) with 41% loss at Day 15 in drought and 32% loss (P= 0.006) in flooding individual treatments but recovered to control levels by Day 30 in plants returned to normal watering (**Figure 3A**). P_n_ was also lower than control in D-F (43% decrease with P= 0.0003) and F-D (36% decrease with P= 0.002) treatments but again recovered at Day 45 (**Figure 3B**).

In barley at 15 days of treatment, net photosynthetic rate (P_n_) was not affected by either drought or flooding treatment, though a significant post-flooding effect was observed (**Figure 3C**). Plants that were exposed to F-D treatment also showed a 44% (P= 0.03) decrease of photosynthesis at Day 30, which recovered when plants were returned to control conditions. Drought treatment imposed following flooding (D-F treatment) did not significantly affect P_n_ of barley (**Figure 3D**).

Both drought and flooding decreased stomatal conductance (g_s_) significantly in wheat, with this effect being increased when stresses were cycled. Stomatal conductance was decreased in wheat at 15 days in both drought and flooding treatments and did not recover at 30 days of recovery period (**Figure 3E**). It was also decreased in drought followed by flooding (D-F) by 74% and flooding followed by drought (F-D) treatments by 78% and at both treatments the significance level was greater than 0.0001 (**Table 2**). Only the D-F treatment recovered to control levels at 45 days. There was also an age-dependent loss of stomatal conductance seen, with control plants also having low g_s_ (**Figure 3F**) and two-way ANOVA results supported the days effects with significant differences (P= 0.02; **Table 2**).

**Table 2:** Results (F and P values) of two-way ANOVA of gas exchange of wheat and barley under cycles of drought and flooding.

Traits	Wheat				Barley			
	Single stress		Successive stress		Single stress		Successive stress	
	F	P	F	P	F	P	F	P
P_n_								
Treatments	14.01	**0.0002**	11.31	**0.0007**	4.510	**0.0258**	5.255	**0.0159**
Days	13.26	**0.0019**	3.796	0.0671	31.42	**<0.0001**	3.576	0.0748
Interaction	3.009	0.0746	7.525	**0.0042**	5.653	**0.0124**	1.807	0.1926
gs								
Treatments	21.04	**<0.0001**	30.75	**<0.0001**	9.777	**0.0012**	92.11	**<0.0001**
Days	6.379	**0.0201**	0.4028	0.5352	1.620	0.2185	33.41	**<0.0001**
Interaction	1.880	0.1785	16.17	**0.0002**	32.73	**<0.0001**	2.798	0.0890

Bold P values indicate significant differences at P< 0.05.

In barley, g_s_ decreased at 15 days of drought but recovered at 30 days, following rewatering. In contrast, g_s_ was not affected during flooding, though a significant post-flooding effect was seen (**Figure 3G**). In plants exposed to F-D treatment, g_s_ was greatly reduced (88%) and did not recover by Day 45. In contrast, D-F treatment had no significant effect on stomatal conductance in barley (**Figure 3H**).

### 3.3 Stomatal density and size changed at F-D double stress in barley, but not in wheat

**Figure 4:**
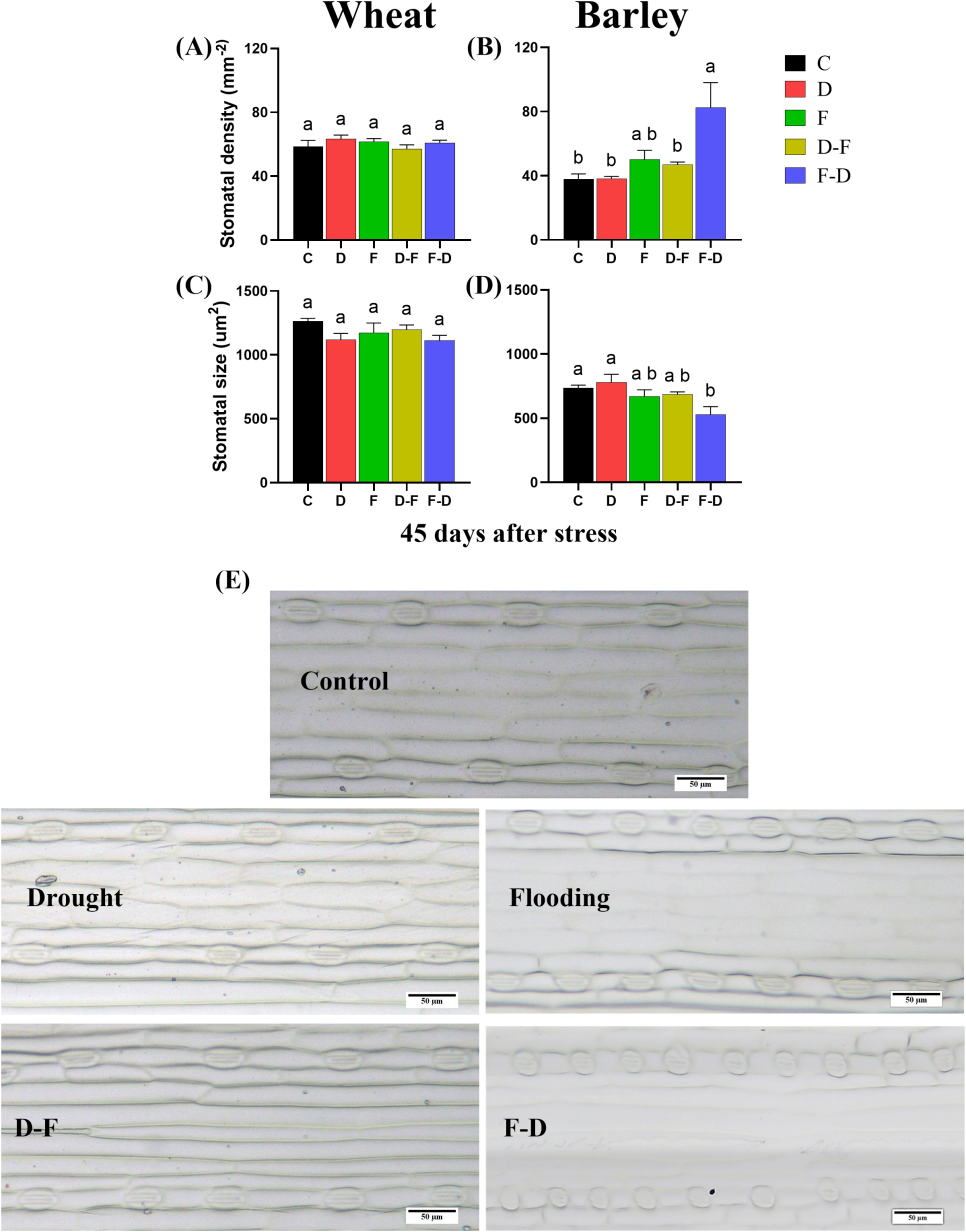
Stomatal characterization of wheat and barley leaves. Leaf samples (4^th^) were harvested at 45 days after stress (59 days-old plant) when all treatment plants were in recovery period. Stomatal density and stomatal size in wheat **(A, C)** and barley **(B, D)** in different treatments. **(E)** Adaxial surface showing distribution of stomata and stomatal size (10x) in barley (Scale bars= 50 µm). Different letters above the bars show significant differences at p< 0.05 (one-way ANOVA/Tukey *post hoc* test). C, control; D, drought; F, flooding; D-F, drought followed by flooding; F-D, flooding followed by drought.

Plants from all treatments were grown to Day 45 after initiation of the stress and leaf samples were harvested for stomatal characterization, when all treatment plants were in recovery period. Stomatal density and size were unchanged in wheat under the different treatments as compared to control (**Figure 4A, C**). In barley however, flooding followed by drought (F-D) treatment caused significant increase of stomatal density, whilst stomatal size was reduced at this treatment (**Figures 4B, D, E**).

### 3.4 Chlorophyll fluorescence in response to cycles of drought and flooding

The maximum quantum yield of Photosystem II (Fv/Fm) is considered as an indicator of plant stress affecting photosynthesis (Maxwell and Johnson, 2000). In this experiment, the only stress treatment to cause a decrease in Fv/Fm was the D-F treatment in wheat, and this completely recovered in normal watering at 30 days (**Supplementary Figure S1B**). In barley, Fv/Fm was insensitive to treatment (**Supplementary Figures S1C, D**). The two-way ANOVA results for Fv/Fm at sequential stress treatments showed significant differences in wheat and no significant differences in barley (**Table 3**).

**Table 3:** Results (F and P values) of two-way ANOVA of chlorophyll fluorescence of wheat and barley under cycles of drought and flooding.

Traits	Wheat				Barley			
	Single stress		Successive stress		Single stress		Successive stress	
	F	P	F	P	F	P	F	P
Fv/Fm								
Treatments	3.318	0.0570	6.694	**0.0067**	0.6207	0.5472	1.881	0.1784
Days	60.81	**0.0001**	1.327	0.2644	15.45	**0.0008**	2.860	0.1063
Interaction	1.574	0.2319	1.502	0.2494	0.05672	0.9450	1.050	0.3684
ΦPSII								
Treatments	0.4163	0.6650	9.562	**0.0017**	1.173	0.3348	4.696	**0.0238**
Days	39.32	**<0.0001**	1.171	0.2944	19.92	**0.0004**	0.004	0.9459
Interaction	0.0171	0.9831	2.706	0.0954	1.654	0.2224	3.662	**0.0476**
NPQ								
Treatments	1.284	0.2979	2.379	0.1184	0.6300	0.5429	0.5019	0.6128
Days	9.628	**0.0054**	0.5842	0.4536	43.11	**<0.0001**	0.6949	0.4143
Interaction	0.5821	0.5675	0.6736	0.5211	0.3780	0.6900	0.8766	0.4316

Bold P values indicate significant differences at P< 0.05.

**Figure 5:**
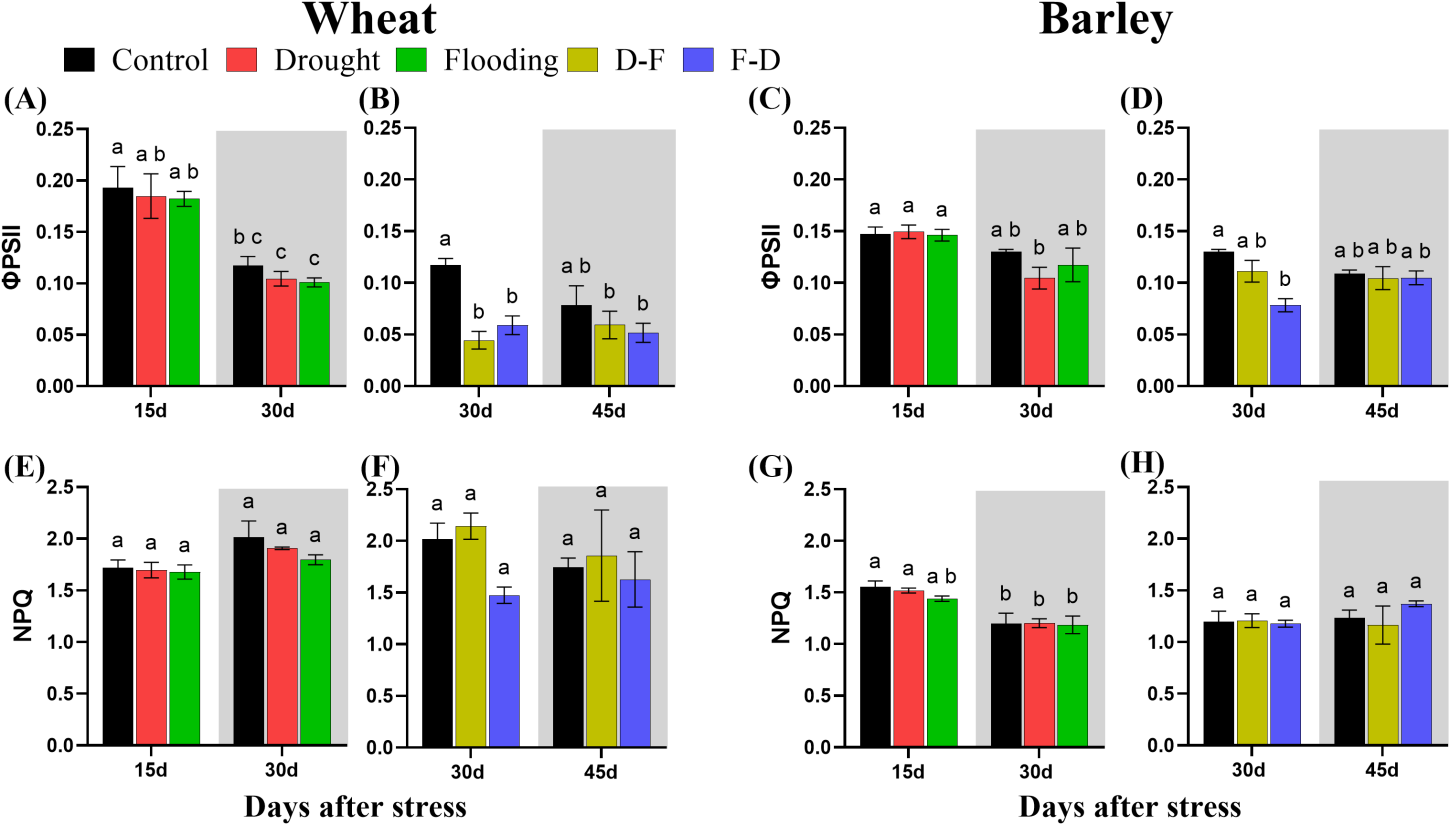
The efficiency of photosystem II [ϕPSII; **(A–D)**] and non-photochemical quenching [NPQ; **(E–H)**] under cycles of drought and flooding in wheat **(A, B, E, F)** and barley **(C, D, G, H)**. Grey areas correspond to recovery. Data were recorded during gas exchange measurements under the same conditions as P_n_ (**Figure 2**). Data are the mean ± SE of 5 biological replicates. Different letters above the bars show significant differences at p< 0.05 (two-way ANOVA/Tukey *post hoc* test). C, control; D, drought; F, flooding; D-F, drought followed by flooding; F-D, flooding followed by drought.

Chlorophyll fluorescence (**Figure 5**) was measured simultaneously with gas exchange (**Figure 3**). Both wheat and barley plants exposed to drought and flooding conditions had no change of Photosystem II efficiency (ϕPSII) as compared to control (**Figures 5A, C**). By contrast, D-F treatment reduced ϕPSII by 62% (P= 0.004) and F-D treatment with 50% loss (P= 0.04) in wheat (**Figure 5B**). But it was only reduced in barley under F-D treatment (**Figure 5D**). However, when these both plants were transferred back to normal watering condition, ϕPSII increased, reaching about the same value as control plants (**Figures 5B, D**).

There was no significant change of non-photochemical quenching (NPQ) under drought and flooding individual treatments and cycles of drought-flooding treatments in either wheat or barley (**Figures 5E–H**; **Table 3**).

### 3.5 Changes of chlorophyll content in response to cycles of drought and flooding

**Figure 6:**
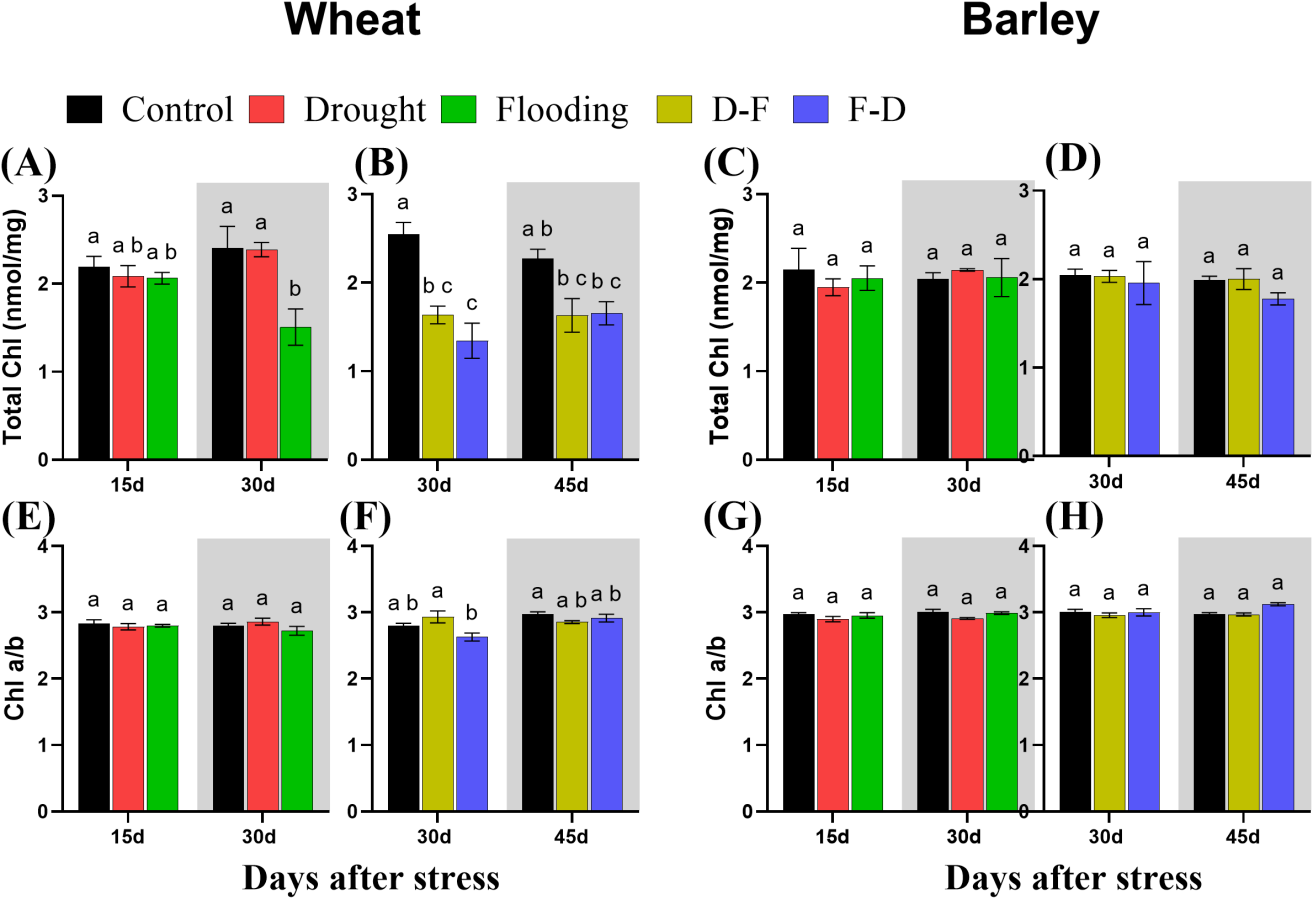
Total chlorophyll content **(A–D)** and chlorophyll a:b ratio **(E–H)** under cycles of drought and flooding in wheat **(A, B, E, F)** and barley **(C, D, G, H)**. Grey areas correspond to recovery. Chlorophyll was extracted from the same leaves used for measuring gas exchange and chlorophyll fluorescence. Data are the mean ± SE of 5 biological replicates. Different letters above the bars show significant differences at p< 0.05 (two-way ANOVA/Tukey *post hoc* test). C, control; D, drought; F, flooding; D-F, drought followed by flooding; F-D, flooding followed by drought.

Chlorophyll content was determined using the same leaves as for measuring gas exchange and chlorophyll fluorescence. In wheat, flooding decreased total chlorophyll content, regardless of when this stress was applied. Total chlorophyll content was decreased in wheat after flooding stress at Day 30 (**Figure 6A**). It was also decreased in D-F (36%) and F-D (47%) treatments and did not recover to control levels by Day 45 (**Figure 6B**). Chlorophyll a/b ratio was unchanged at both single and double stress as compared to control, though there was a significant difference of chlorophyll composition between D-F and F-D treatments in wheat (**Figure 6E, F**). By contrast, in barley, there was no changes in chlorophyll content or composition in response to single drought or flooding and D-F or F-D stress (**Figure 6C, D, G, H**). The two-way ANOVA results show there was significant differences for chlorophyll content in wheat and no significant differences in barley (**Table 4**).

**Table 4:** Results (F and P values) of two-way ANOVA of chlorophyll content of wheat and barley under cycles of drought and flooding.

Traits	Wheat				Barley			
	Single stress		Successive stress		Single stress		Successive stress	
	F	P	F	P	F	P	F	P
Total Chl								
Treatments	8.186	**0.0032**	19.67	**<0.0001**	0.0551	0.9465	0.6521	0.5335
Days	0.0100	0.9212	0.0060	0.9391	0.0668	0.7988	0.4917	0.4927
Interaction	5.880	**0.0115**	1.779	0.2049	0.4454	0.6471	0.1400	0.8703
Chl a/b								
Treatments	0.9958	0.3855	2.172	0.1429	3.444	0.0500	2.774	0.0891
Days	0.1188	0.7336	2.172	**0.0294**	0.9779	0.3335	0.9806	0.3352
Interaction	1.412	0.2650	3.980	**0.0370**	0.1078	0.8983	1.758	0.2008

Bold P values indicate significant differences at P< 0.05.

### 3.6 Effects of cycles of drought and flooding on total nitrogen and carbon content of wheat and barley plants

**Figure 7:**
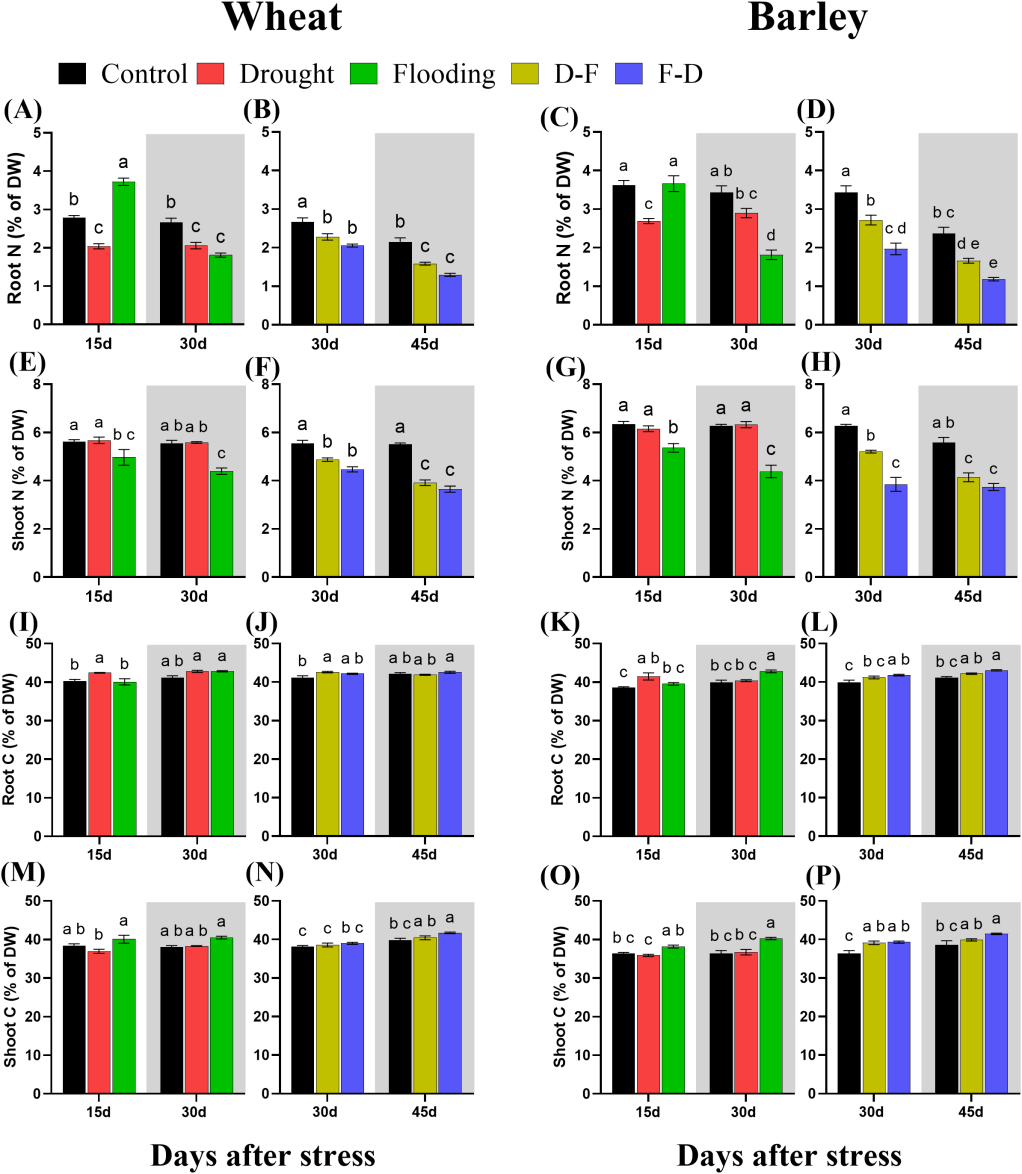
Changes in root nitrogen content in wheat **(A, B)** and barley **(C, D)**; shoot nitrogen content in wheat **(E, F)** and barley **(G, H)**; root carbon content in wheat **(I, J)** and barley **(K, L)**; shoot carbon content in wheat **(M, N)** and barley **(O, P)** under cycles of drought and flooding. Grey areas correspond to recovery. Data are the mean ± SE of 5 biological replicates. Different letters above the bars show significant differences at p< 0.05 (two-way ANOVA/Tukey *post hoc* test). C, control; D, drought; F, flooding; D-F, drought followed by flooding; F-D, flooding followed by drought.

All treatments affected nitrogen content and distribution in both plant species. Total nitrogen, expressed as a percentage of dry matter, decreased in wheat roots in response to drought and increased at Day 15 in response to flooding, though decreased subsequently after the stress was removed, at Day 30 (**Figure 7A**). There was no change of shoot nitrogen under drought in wheat but flooding reduced shoot nitrogen content in this species (**Figure 7E**). Meanwhile, both D-F and F-D treatments decreased nitrogen content in wheat roots and shoots, and this did not recover after the stress period at Day 45 (**Figure 7B, F**). In Barley, drought decreased nitrogen content in roots, but flooding did not change nitrogen content at Day 15, though a significant and substantial reduction was observed after recovery at Day 30 (**Figure 7C**). However, nitrogen content of shoots in barley was not affected by drought but was reduced in response to flooding (**Figure 7G**). Similarly, D-F and F-D treatments affected nitrogen content in both root and shoot of barley, which did not recover after the stress period at Day 45 (**Figure 7D, H**).

There was no change of carbon content in wheat, except in response to drought and drought following flooded in roots. In barley, drought increased root carbon content and all other treatments induced an increase of this content in both roots and shoots with an even higher content at recovery period under flood and F-D stress (**Figure 7**). In wheat, drought increased the carbon content of roots, though this recovered in normal watering by Day 30, while flooding did not change the carbon content in roots (**Figure 7I**). Meanwhile, there was no change of carbon content in shoots of wheat at drought or flooding though significant decrease was observed as compared to flooded wheat (**Figure 7M**). However, D-F stress increased carbon content followed by recovery, while F-D stress did not change the content in roots of wheat and there was no change of carbon content in shoots of wheat under both D-F and F-D treatments (**Figure 7J, N**). In barley, drought increased carbon content of root with a recovery at Day 30 and flooding did not change the content at stress period but increased by 7.2% significantly (P= 0.006) at recovery, Day 30 (**Figure 7K**). By contrast, drought had no effect on the shoot carbon, but flooding increased carbon content in the shoot of barley as compared to droughted barley and continued after the stress, Day 30 (**Figure 7O**). Carbon content of roots was increased in response to F-D, as compared to control, in barley, but not in D-F treatments with even a higher content after the stress at Day 45 (**Figure 7L**). Besides, both D-F (7.47%) and F-D (7.96%) treatments increased the carbon content in the shoots of barley, but only F-D stressed barley had high carbon content at recovery period, Day 45 (**Figure 7P**). However, cycles of drought and flooding induced high C:N ratios in both wheat and barley species under all treatments (**Supplementary Figure S2**). The two-way ANOVA results show there were significant differences for C and N content in both wheat and barley (**Supplementary Table S1**).

## 4 Discussion

Episodes of extreme weather are projected to increase and fluctuate between extreme dry and wet conditions. To enhance stress tolerance in crop plants, particularly cereals, we need to better understand the traits possessed by tolerant plants. In this study, a barley cultivar, Chariot- generally considered a relatively stress tolerant cereal species under different environmental conditions, and a wheat cultivar, Gleam- a high yielding adaptable variety that can adapt to different environmental stress, were examined, with the hypothesis that differences between these two plant species might exist in their responses to cycles of drought and flooding. Our results show clear contrasts between the two cultivars studied, indicating that successive stresses increase the sensitivity by the exposure to a first stress, and that barley is less sensitive than wheat. Inhibition in barley could mostly be explained by stomatal limitation of photosynthesis, whilst in wheat, non-stomatal limitations were important.

In this study, drought or flooding were applied as single stress (15 days) or as sequential stresses, in which one stress was followed by the other (15 days each, total 30 days). While this introduces a difference in total stress duration between single and sequential stresses, the experimental design was set up to reflect ecologically relevant scenarios, where plants encounter temporally separated but contrasting water stresses. To enable us to isolate the effects of stress sequence and transition (drought to flooding or flooding to drought) on photosynthetic responses, each individual stress duration was standardized to 15 days. Although the extended duration of the sequential stress treatments may contribute to cumulative effects, our primary focus was on how the transition between stress types influence physiological performance. This approach is supported by previous findings emphasizing the significance of both stress duration and sequence stimulating plant responses to sequential abiotic stress. For example, Ru et al. (2022) showed that prior exposure to drought can enhance tolerance to subsequent heat stress in maize through mechanisms such as improved photosynthetic efficiency and by activating stress memory in plants under water stress (Luis de la Fuente et al., 2023). By comparing responses in wheat and barley, we further examined whether prior exposure to one stress stimulates the response to a subsequent, contrasting stress- highlighting possible mechanism or recovery effects specific to species and stress order. These insights contribute to a broader understanding of how cereals cope with dynamic and fluctuating environmental conditions, particularly in the context of climate change.

Previously, only a few studies have examined responses to cycles of drought and flooding. Zhu et al. (2020) showed the cumulative effects of drought followed by flooding (D-F) reduced photosynthesis followed by recovery and compensation in rice. They also observed both synergistic or antagonistic effects on photosynthesis in previously droughted rice following flooding at different drought or flooding intensities. To be best of our knowledge, no work has been reported on photosynthetic responses in plants exposed to flooding followed by drought (F-D) and importantly, photosynthetic responses of relatively stress tolerant species like barley have not so far been examined under drought-flood water stress. Though some studies have focused on yield loss in wheat (Dickin and Wright, 2008), cotton (Qian et al., 2020) and cabbage (Barber and Müller, 2021) under drought-flood stress, photosynthetic responses, that may be important to find out tolerance traits under double water stress have not been described. In our experiments, two-week-old wheat and barley plants were studied under drought, flooding and D-F or F-D treatments. We chose in this study to measure photosynthetic parameters along with growth, as a previous study on barley photosynthesis showed a significant correlation between photosynthetic efficiency, especially maximum quantum yield of photosystem II, and yield related traits that suggests the improving crop yield through optimizing photosynthetic light use efficiency (Gao et al., 2024).

Wheat plants exposed to a single stress, either drought or flooding, experienced a decrease of photosynthetic capacity (**Figure 3A**), and growth of droughted wheat was reduced post-flooding (**Figures 2A, E**, grey area). By contrast, neither drought nor flooding as a single stress inhibited the growth of barley (**Figures 2C, G**), with flooding increasing root growth post-stress, Day 30 (**Figure 2C** grey area, **Supplementary Figure S3**). There was no decrease of maximum photosynthetic rate during stress in barley, though this was inhibited post-flooding (**Figure 3C**). Overall, results are consistent with this species generally being more stress tolerant than wheat. The increasing root:shoot ratio in barley following flooding, Day 30 (**Figure 2K**, grey area) indicates that this had the greatest impact on plant development, which might be expected to affect subsequent stress responses. Yang et al. (2004) reported that extensive root systems can help maintain photosynthetic rate by supplying water, nutrients and plant hormones under stress conditions. The reduction of growth and photosynthesis by single- drought (Ahmad et al., 2018; Zhao et al., 2020) or flooding (Shao et al., 2013) stress has been reported previously in wheat and reduction in the photosynthetic capacity depends on the intensity of water stress (Chen et al., 2025). Also, an increase in photosynthetic capacity in tolerant barley varieties induced by single- drought has also been observed (Harb et al., 2020), and the tolerance of flooded barley measured by Fv/Fm (Langan et al., 2024) but the post-flooding inhibition of photosynthesis in this species has not to our knowledge been reported before. Here, we observed that barley photosynthetic capacity is less sensitive to single- drought or flooding stress than wheat. We assessed physiological reasons associated with these contrasting responses by investigating chlorophyll fluorescence, chlorophyll content and C-N content. ϕPSII was unaffected by drought and flooding treatments in both species (**Figures 5A, C**). This contrasts with P_n,_ which was inhibited in wheat in both stresses (**Figure 3A**). This implies that changes in P_n_ are a result mainly of stomatal closure limiting CO_2_ supply, while ϕPSII is maintained either through acclimation of the photosynthetic apparatus (reducing PSII capacity) or simply through an increase in photorespiration. The loss of chlorophyll in wheat after flooding (**Figure 6A**, grey area) may reflect an acclimation response in this case, correlating with a loss of leaf N, whilst C did not change (**Figures 7E, M**, grey area). In contrast, in drought or flooded barley there was no reduction of photosynthesis or ϕPSII, and no change in chlorophyll or leaf N. There was an increase in shoot C in flooded barley, which continued after the stress, Day 30 (**Figure 7O**) and is suggested to reflect the accumulation of sugars in leaves (Martínez-Alcántara et al., 2012) which may help the osmotic adjustment of stressed barley.

Droughted wheat which was then exposed to flooding (D-F) showed reduced stomatal conductance (**Figure 3F**) which was similar to the findings of experiments on rice (Zhu et al., 2020). In our experiment, the inhibited stomatal conductance (g_s_) in the early droughted wheat (**Figure 3E**) was followed by a greater inhibition of g_s_ following subsequent exposure to flooding (**Figure 3F**), which contributed to a decreased rate of photosynthesis (**Figure 3B**). At the same time, D-F treatment reduced ϕPSII, accompanied by a loss of chlorophyll, neither of which were affected by drought alone (**Figures 5B**, **6B**). Alongside this loss of photosynthesis, wheat growth was highly retarded following D-F treatment, as compared to drought alone (**Figures 2B, F**). This suggests D-F stress induces some non-stomatal factors that limit photosynthesis and growth in wheat. C-N content (**Figure 7**) reflects a loss of nitrogen, which may affect growth. The loss of N in shoots, without any change of carbon content, may contribute to the loss of photosynthesis in D-F stress in wheat. Therefore, we found that the effect of flooding on the previously droughted plants was continued and aggravated in wheat, as also previously observed in wheat (Schweiger et al., 2023). The prior inhibition of photosynthesis of single-droughted wheat could be explained by stomatal closure, but after subsequent flooding, non-stomatal factors were added, which were probably linked to the reduced PSII efficiency and chlorophyll content.

In contrast to wheat, when barley was flooded after drought, there was a recovery of stomatal conductance, whilst P_n_ was unaffected in either drought or D-F treatments (**Figure 3**). In addition, there was no change of ϕPSII (**Figure 5D**) or chlorophyll content (**Figure 6D**) in either treatment. There was no effect of D-F treatment on root growth, but the shoot growth was retarded (**Figures 2D, H**). This effect on growth was also reflected in their C-N content, showing that there was no change of N-content in the shoots in droughted barley, but a loss of N, accompanied by an increase in C- content in the shoot of D-F barley (**Figures 7H, P**). Therefore, we conclude that barley can retain stomatal conductance and photosynthesis under D-F stress whilst decreasing shoot growth, increasing root:shoot ratio. The increased leaf C- content suggests an accumulation of sugar in shoots.

For the F-D treatment in wheat, stomatal closure was greater than seen for drought as a single stress (**Figures 3E, F**) although there was no greater inhibition of photosynthesis (**Figures 3A, B**). This is accompanied by a similar downregulation ϕPSII in the F-D treatment (**Figure 5B**). This suggests a downregulation of electron transport to match production of ATP and NADPH to the decreased CO_2_ assimilation (Lu and Zhang, 1999). In addition, the loss of chlorophyll content under F-D stress may contribute to the low rate of photosynthesis (**Figure 6B**). Though there was no effect on their growth exposed to prior flooding, after F-D stress (Day 45) the growth was greatly retarded (**Figures 2B, F**). Therefore, the inhibition induced by flooding is continued when plants are exposed to subsequent drought. Like the D-F treatment, our observations showed the involvement of non-stomatal factors decreasing photosynthesis and growth under F-D stress conditions in wheat.

In barley, the F-D treatment, unlike the D-F treatment, induced a large inhibition of g_s_, accompanied by an inhibition of the rate of photosynthesis (**Figures 3D, H**), together with the downregulation of ϕPSII followed by recovery (**Figure 5D**). There was no accompanying change of chlorophyll content (**Figure 6D**). However, they retained their root growth under F-D stress (**Figure 2D**), but shoot growth was retarded, giving a higher root/shoot ratio, similar to that in D-F stressed barley (**Figures 2H, L**). When previously flooded barley was droughted, the inhibition of g_s_ was greater than that caused by drought as a single stress (**Figure 3G**) which did not fully recover when plants returned to normal watering conditions (**Figure 3H**), although P_n_ recovered (**Figure 5D**). The response of g_s_ may be explained by developmental differences in leaves formed during flooding (**Figure 4**). Leaves formed during the initial flooding showed a higher stomatal density, but these stomata were smaller than those formed under control conditions. A similar developmental effect has previously been seen in barley grown at a high CO_2_ concentration (Haworth et al., 2023; Kreuzwieser et al., 2004). Moreover, F-D stressed barley had increased C-content in both roots and shoots (**Figures 7L, P**) possibly reflecting an accumulation of carbohydrates in their tissue. Overall, we conclude that previous flooding increases barley sensitivity to subsequent drought, exacerbating the post-flooding effect seen when plants are returned to normal watering. Further study is required to understand the stomatal limitation to photosynthesis of barley in F-D stress.

## 5 Conclusion

**Figure 8:**
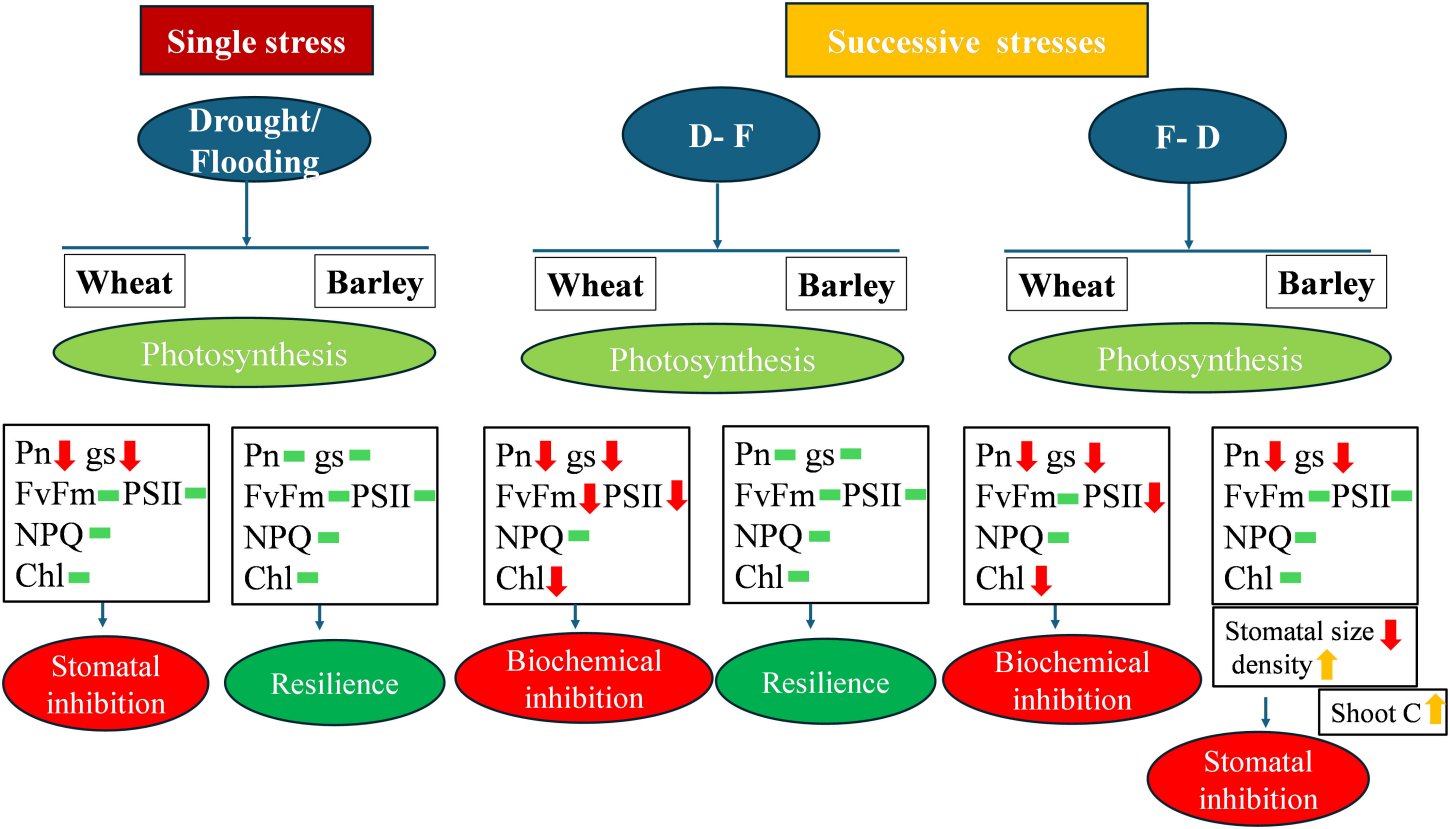
Schematic diagram model of differential physiological responses demonstrating biochemical limitation to photosynthesis in wheat and stomatal limitation in barley. Red arrows indicate ‘decrease’, yellow arrows indicate ‘increase’ and green bar indicate ‘no significant change‘.

The present study has investigated the physiological responses of wheat and barley to cycles of drought and flooding and shown contrasting responses in the two species (**Figure 8**). We observed that exposure to the first stress stimulated the inhibition of photosynthesis induced by further stress. Importantly, preceding exposure to flooding increased the inhibition of photosynthesis induced by subsequent drought in both wheat (36%) and barley (44%). Barley is relatively more tolerant to successive water stresses retaining photosynthetic capacity and PSII efficiency following single as well as double water stress, with only F-D double stress inhibiting photosynthesis and only via a stomatal limitation. The greater root growth (79% increase) of barley may be important in this tolerance. By contrast, wheat may increase its chances of survival by having the flexibility to reengineer the leaf, even if it is unable to maintain its growth and final yield. These findings have important implications for agricultural production in sequential water stressed environments. The relative successive drought and flood resilience of barley suggests it could be a more sustainable crop choice under climate change with frequent water stress. It could potentially ensure more stable yields leading to food security. Future research should focus on underlying photosynthetic mechanisms of barley under sequential flood-drought conditions and yield variations to optimize crop performance.

## Data Availability

The raw data supporting the conclusions of this article will be made available by the authors, without undue reservation.
