# Supplementary material for: Divergent effects of successive drought and flooding on photosynthesis in wheat and barley
Source: Front Plant Sci. 2025 Aug 25;16:1603355. doi: 10.3389/fpls.2025.1603355 (PMC12415061; doi:10.3389/fpls.2025.1603355)
Supplement: Supplementary file 1 [file DataSheet1.docx]

## ***Supplementary Material***

1. **Supplementary Figures**


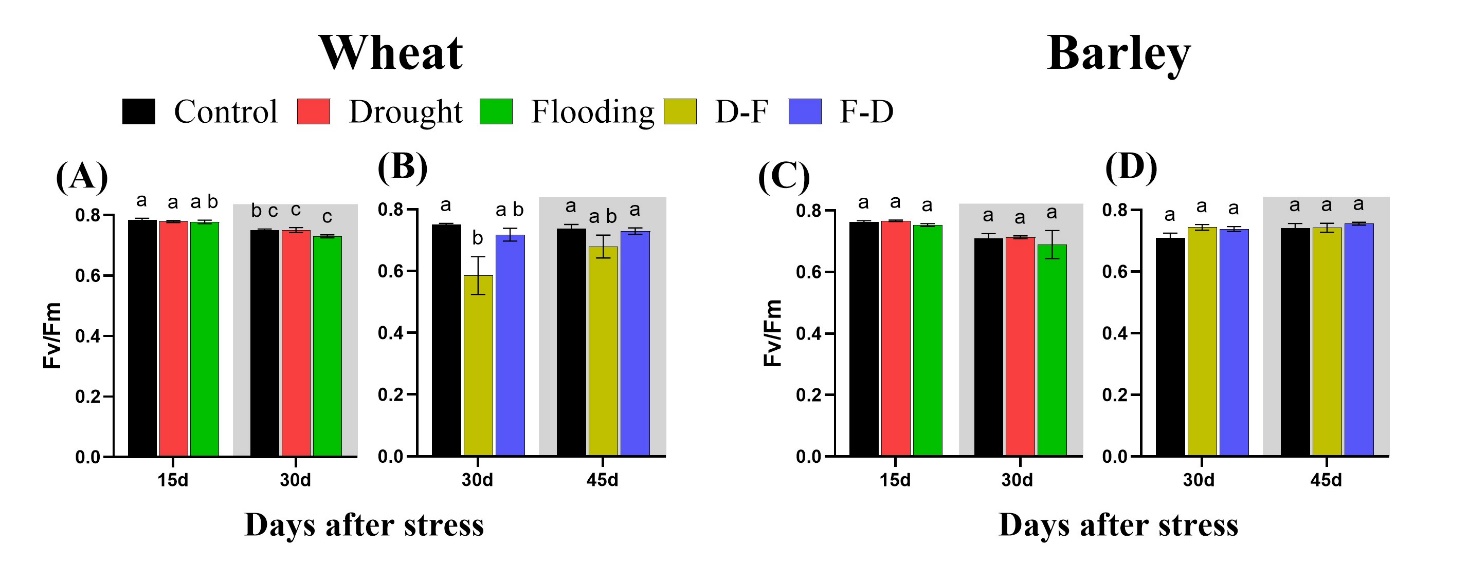


**Supplementary Figure S1.** Ratio of Fv/Fm under cycles of drought and flooding in wheat (A, B) and barley (C, D). Grey areas correspond to recovery. Data from the same plants as used in Fig. 4.


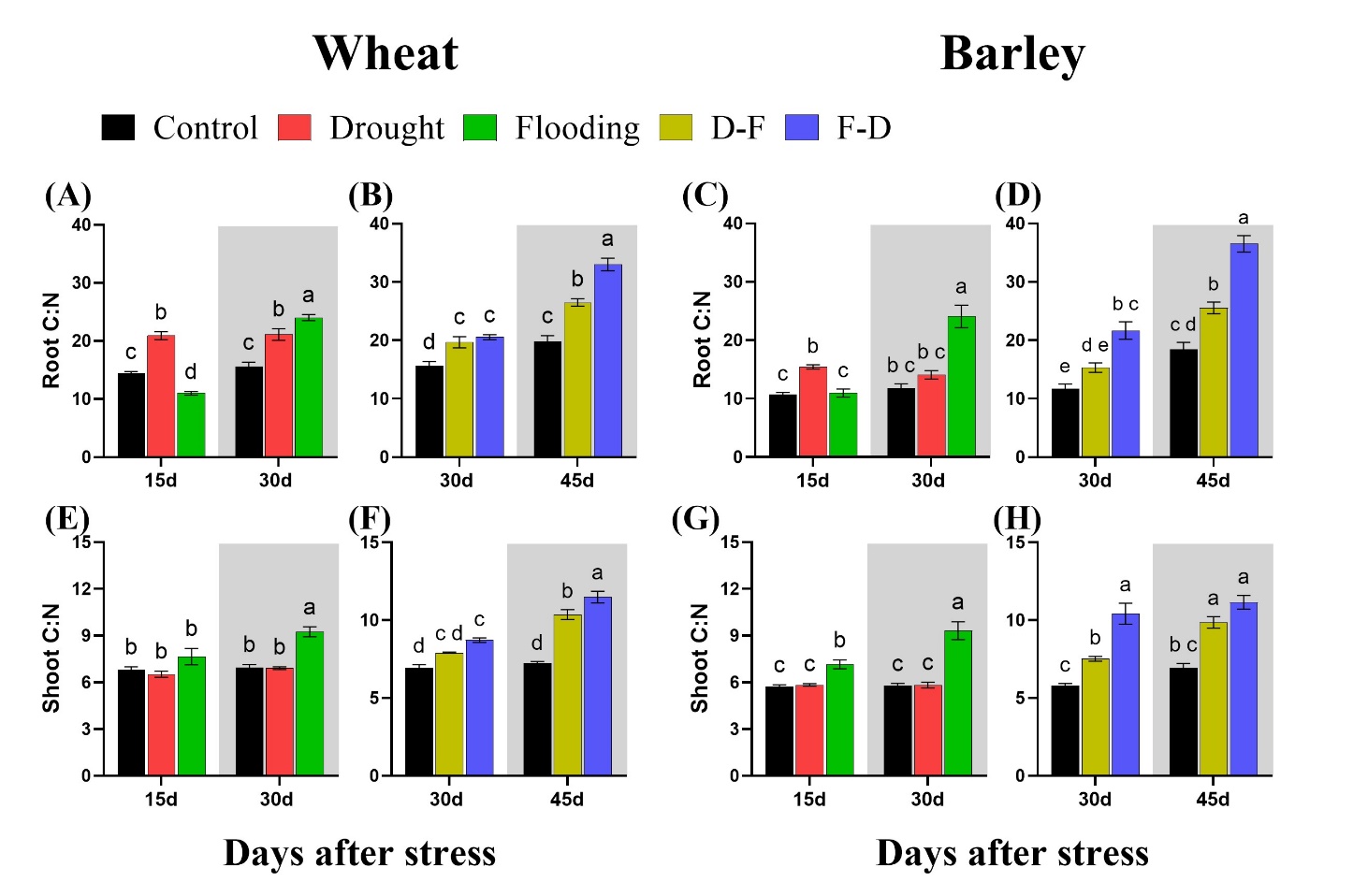


**Supplementary Figure S2.** Changes to C:N ratio of root and shoot under cycles of drought and flooding in wheat (A, B & E, F) and barley (C, D & G, H). Grey areas correspond to recovery. Values calculated from data in Fig. 6.


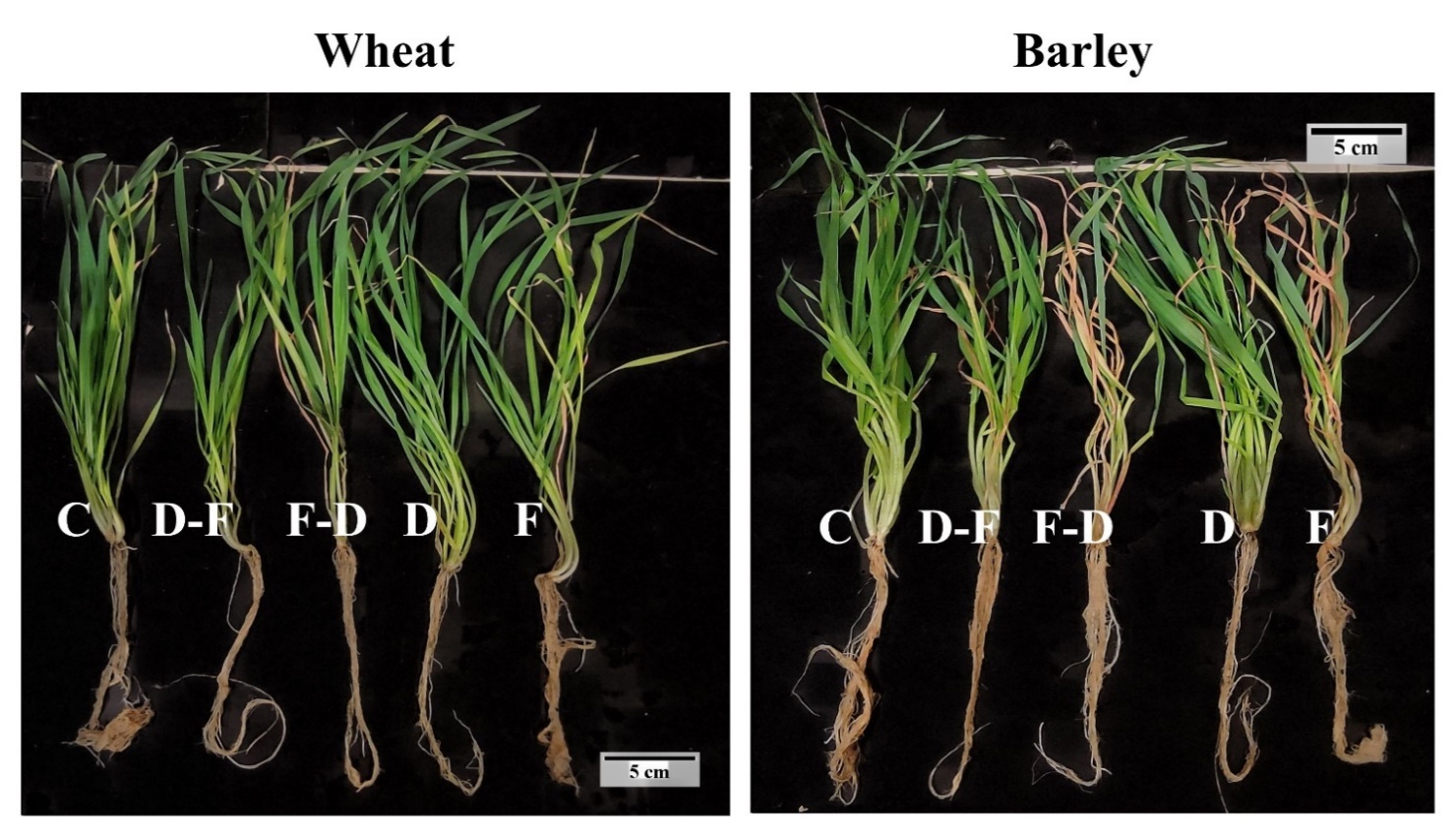


**Supplementary Figure S3.** Images of wheat (left) and barley (right) 45 days after stress under cycles of drought and flooding. C, control; D, drought; F, flooding; D-F, drought followed by flooding; F-D, flooding followed by drought.

1. **Supplementary Table**

**Table S1:** Results (F and P values) of two-way ANOVA of C and N content and C/N ratios of wheat and barley under cycles of drought and flooding.

| Traits | Wheat | | | | Barley | | | |
| --- | --- | --- | --- | --- | --- | --- | --- | --- |
|  | Single stress | | Successive stress | | Single stress | | Successive stress | |
|  | F | P | F | P | F | P | F | P |
| Root N content | | | | | | | | |
| Treatments | 52.44 | **<0.0001** | 49.04 | **<0.0001** | 19.75 | **<0.0001** | 53.32 | **<0.0001** |
| Days | 107.3 | **<0.0001** | 114.7 | **<0.0001** | 27.92 | **<0.0001** | 84.82 | **0.0001** |
| Interaction | 88.25 | **<0.0001** | 1.444 | 0.2557 | 29.77 | **<0.0001** | 0.7620 | 0.4777 |
| Shoot N content | | | | | | | | |
| Treatments | 28.70 | **<0.0001** | 112.7 | **<0.0001** | 54.46 | **<0.0001** | 72.05 | **<0.0001** |
| Days | 5.004 | **0.0357** | 52.28 | **<0.0001** | 5.370 | **0.0293** | 18.36 | **0.0003** |
| Interaction | 2.025 | 0.1559 | 11.63 | **0.0003** | 7.565 | **0.0028** | 3.647 | **0.0414** |
| Root C content | | | | | | | | |
| Treatments | 8.913 | **0.0013** | 3.832 | **0.0359** | 8.275 | **0.0018** | 16.17 | **<0.0001** |
| Days | 13.15 | **0.0013** | 0.7735 | 0.3878 | 7.648 | **0.0108** | 18.13 | **0.0003** |
| Interaction | 3.906 | **0.0340** | 4.392 | 0.0237 | 8.803 | **0.0014** | 0.1457 | 0.8652 |
| Shoot C content | | | | | | | | |
| Treatments | 12.47 | **0.0002** | 6.200 | **0.0067** | 21.91 | **<0.0001** | 11.96 | **0.0002** |
| Days | 1.220 | 0.2804 | 42.18 | **<0.0001** | 5.971 | **0.0223** | 11.62 | **0.0023** |
| Interaction | 1.058 | 0.3628 | 0.9228 | 0.4111 | 2.033 | 0.1529 | 0.8736 | 0.4303 |
| Root C/N | | | | | | | | |
| Treatments | 42.56 | **0.0001** | 58.28 | **<0.0001** | 21.14 | **<0.0001** | 75.30 | **<0.0001** |
| Days | 77.39 | **<0.0001** | 127.7 | **<0.0001** | 29.10 | **<0.0001** | 126.3 | **<0.0001** |
| Interaction | 55.58 | **<0.0001** | 12.30 | **0.0002** | 32.00 | **<0.0001** | 6.377 | **0.0060** |
| Shoot C/N | | | | | | | | |
| Treatments | 24.72 | **<0.0001** | 88.07 | **<0.0001** | 49.58 | **<0.0001** | 63.45 | **<0.0001** |
| Days | 10.26 | **0.0040** | 92.96 | **<0.0001** | 10.23 | **0.0039** | 19.27 | **0.0002** |
| Interaction | 4.188 | **0.0281** | 16.81 | **<0.0001** | 9.459 | **0.0009** | 2.231 | 0.1292 |

Bold P values indicate significant differences at P< 0.05.
